# Butyrate Conditions Human Dendritic Cells to Prime Type 1 Regulatory T Cells *via* both Histone Deacetylase Inhibition and G Protein-Coupled Receptor 109A Signaling

**DOI:** 10.3389/fimmu.2017.01429

**Published:** 2017-10-30

**Authors:** Maria M. M. Kaisar, Leonard R. Pelgrom, Alwin J. van der Ham, Maria Yazdanbakhsh, Bart Everts

**Affiliations:** ^1^Department of Parasitology, Leiden University Medical Center (LUMC), Leiden, Netherlands; ^2^Faculty of Medicine, Department of Parasitology, Universitas Indonesia, Jakarta, Indonesia

**Keywords:** short-chain fatty acids, butyrate, dendritic cells, type 1 regulatory T cells, histone deacetylases, G-coupled protein receptor 109A, retinaldehyde dehydrogenase, retinoic acid

## Abstract

Recently, it has become clear that short-chain fatty acids (SCFAs), and in particular butyrate, have anti-inflammatory properties. Murine studies have shown that butyrate can promote regulatory T cells *via* the induction of tolerogenic dendritic cells (DCs). However, the effects of SCFAs on human DCs and how they affect their capacity to prime and polarize T-cell responses have not been addressed. Here, we report that butyrate suppresses LPS-induced maturation and metabolic reprogramming of human monocyte-derived DCs (moDCs) and conditions them to polarize naive CD4^+^ T cells toward IL-10-producing type 1 regulatory T cells (Tr1). This effect was dependent on induction of the retinoic acid-producing enzyme retinaldehyde dehydrogenase 1 in DCs. The induction of retinaldehyde dehydrogenase activity and Tr1 cell differentiation by butyrate was dependent on simultaneous inhibition of histone deacetylases and signaling through G protein-coupled receptor 109A. Taken together, we reveal that butyrate is a potent inducer of tolerogenic human DCs, thereby shedding new light on the cellular and molecular mechanisms through which SCFAs can exert their immunomodulatory effects in humans.

## Introduction

Dendritic cells (DCs) play a crucial role in the development of adaptive immune responses during infections and inflammatory diseases, as well as in the regulation of immune homeostasis during steady state, by governing the activation and maintenance of T-cell responses. In response to many viral and bacterial infections, DCs promote the generation of immune responses that are dominated by CD4^+^ T helper 1 (Th1) cells and cytotoxic CD8^+^ T cells. By contrast, fungal and parasitic worm infections are predominantly associated with Th17 and Th2 responses, respectively. In addition to these effector responses, DCs can be instructed to become tolerogenic and promote regulatory T cells (Tregs) responses, a process that is crucial for maintenance of immune homeostasis and control of autoimmune disorders and allergies (1–3).

Over the years, there has been a growing appreciation that microbiota are central players in the education and maintenance of a well-balanced immune system. Among the various mechanisms through which intestinal microbiota have been described to modulate the immune system, the production of short-chain fatty acids (SCFAs) is a major one (4). SCFAs are organic fatty acids with acyl chains consisting of 1–6 carbon atoms (C1–C6) that are the fermentation products of non-digestible polysaccharides by gut microbiota. Acetate (C2), propionate (C3), and butyrate (C4) are among the most abundant species found in the intestine (5). Given their ability to be transported into the circulation, SCFAs can exert functions in organs distal to the intestine (2, 4, 6, 7). In line with this, SCFAs have beneficial effects on a broad range of inflammatory diseases in animal models of inflammatory bowel disease, colitis, asthma, obesity, and arthritis (2, 4, 8, 9).

Short-chain fatty acids have diverse functions depending on the tissue or cell type involved. For instance, SCFAs are crucial for the maintenance of intestinal epithelium physiology by regulating the cellular turnover and barrier functions. SCFAs can also regulate the activation, recruitment and differentiation of immune cells, including neutrophils, DCs, macrophages, and T lymphocytes. In general, SCFAs have anti-inflammatory effects on immune cells. For instance, SCFAs reduce expression of pro-inflammatory cytokines such as tumor necrosis factor (TNF)-α, IL-6 and IL-12 by macrophages, and DCs. In addition, SCFAs, in particular butyrate, can condition murine DCs to promote the differentiation and expansion of Tregs. SCFAs can additionally act on T cells directly, resulting in reduced proliferation and polarization toward a regulatory phenotype (10–12).

Two main mechanisms have been described thus far through which SCFAs can modulate immune cell function. SCFAs can affect immune cells *via* signaling through specific G protein-coupled receptors (GPRs). The most well-characterized SCFAs-sensing GPRs are GPR41, GPR43, and GPR109A (5, 13, 14). In addition, following transport across the plasma membrane *via* monocarboxylate transporter Slc5a8 (15–17), propionate and butyrate can act as inhibitors of histone deacetylase (HDAC) 1 and 3. HDACs together with histone acetylase (HATs) control histone acetylation, which plays a key role in epigenetic regulation of gene expression by serving as a switch between permissive (*via* HAT-induced acetylation) and repressive chromatin (through HDAC-driven deacetylation). While inhibition of HDAC activity can have a wide range of effects including changes in gene expression, chemotaxis, differentiation, proliferation, and apoptosis (9, 11, 18), studies on immune cells have linked HDAC inhibition by SCFAs primarily to suppression of inflammatory responses (19–22). Finally, SCFAs can also act as direct substrates for metabolic processes in cells. For instance, butyrate is known to be a major energy source for gut epithelium (23). However, whether SCFAs also feed into core metabolic pathways of immune cells in a similar manner to regulate their bioenergetic status and whether this has an immunomodulatory effect still needs to be investigated.

Despite the advances in the field, there is still an incomplete understanding of the mechanisms through which SCFAs promote tolerogenic DCs and how these DCs drive Tregs. While one study found that butyrate-driven Treg cell induction by murine DCs is dependent on signaling through GPR109A (13), others have refuted this (16, 24). These latter studies instead implicated the requirement for transport through Slc5a8 and subsequent inhibition of HDAC activity in promoting tolerogenic murine DCs. These butyrate-conditioned murine DCs were found to have increased expression of known immunosuppressive enzymes retinaldehyde dehydrogenase (RALDH) 2 and indoleamine-pyrrole 2,3-dioxygenase (IDO) (16). However, whether RALDH and/or IDO were important in tolerance induction by these DCs was not assessed. Importantly, to date, there has only been a single study assessing the effects of SCFAs on human DCs, in which particularly butyrate was found to suppress LPS-induced maturation (14). Yet, whether or how SCFAs can condition human DCs to prime Tregs remains to be addressed. Given these inconsistencies in murine literature and the paucity in our understanding of how SCFAs affect the functional properties of human DCs, we here set out to assess whether and through which molecular mechanisms SCFAs affect T-cell polarization by human DCs. We find that butyrate through a combination of signaling *via* GPR109A and HDAC inhibition drives retinaldehyde dehydrogenase 1 (RALDH1) expression in human DCs which licenses them to prime type 1 regulatory T cells (Tr1). This provides important new insights into the cellular and molecular mechanisms through which SCFAs can exert their immunomodulatory effects in humans.

## Materials and Methods

### Ethics Statement

Human monocytes and T cells were obtained from blood that was donated to the Bloodbank (Sanquin, Amsterdam) by healthy volunteers. The donated material was processed and analyzed anonymously. As such, not ethical approval was required for these studies.

### Human DC Culture, Stimulation, and Analysis

Monocytes were isolated from venous blood and differentiated into moDCs as described previously (25). In brief, monocytes were isolated using CD14 MACS beads (Miltenyi) according to the manufacturer’s recommendations, routinely resulting in a monocyte purity of >95%. Monocytes were subsequently cultured in RPMI medium supplemented with 10% FCS, 50 ng/mL human rGM-CSF (Invitrogen), and 25 U/mL human rIL4 (R&D Systems) for 6 days to differentiate them into a homogeneous population of CD14 low, CD1a^+^ monocyte-derived DCs (moDCs) (26). On day 6, immature DCs were left untreated or were stimulated with 2 mM SCFAs, namely: acetate, butyrate (both sigma-Aldrich, kind gift from Dr. Martin Giera) or propionate (Sigma-Aldrich); 2.5 µM vitamin D3 (Sigma-Aldrich), trichostatin A (TSA) (100 ng/mL), niacin (2 mM) (Sigma-Aldrich), soluble egg antigens (SEA) (50 µg/mL), and IFN-γ (1,000 U/mL). SEA was prepared as previously described (27). All stimulations were done in the presence of 100 ng/mL ultrapure LPS (*E. coli* 0111 B4 strain, InvivoGen, San Diego, CA, USA), unless indicated otherwise. The DCs were incubated with 10 µM RALDH inhibitor diethylaminobenzaldehyde (DEAB) (Stem Cell Technologies) in the indicated conditions. After 48 h of stimulation, surface expression of costimulatory molecules was determined by flow cytometry (FACS-Canto, BD Biosciences, Breda, The Netherlands) using the following antibodies: CD14 HV450 (MΦP9), CD86 FITC (2331 FUN-1), CD40 APC (5C3), CD80 Horizon V450 (L307.4), CD274/PDL1 PE-Cy7 (MIH1) (all BD Biosciences), HLA-DR APC-eFluor 780 (BL6), CD273/PDL2 PE (MIH18) (eBioscience, San Diego, CA, USA), CD83 PE (HB15e), CD1a PE (BL6) (all Beckman Coulter, Fullerton, CA, USA), and latency-associated peptide (LAP) APC (TW4-2FB) (BioLegend). In addition, 1 × 10^4^ matured moDCs were cocultured with 1 × 10^4^ CD40L-expressing J558 cells. Supernatants were collected after 24 h, and the concentration of IL-10 (Sanquin) and IL-12p70 (using mouse anti-human IL-12, clone 20C2 and biotinylated mouse-anti-human IL-12 clone 8.6, both BD Bioscience) was determined by ELISA.

### Aldefluor Assay

Aldefluor kit (Stemcell Technologies) was used, according to the manufacture’s protocol, to determine RALDH activity.

### Histone 3 (H3) and H4 Acetylation by Flow Cytometry

H3 and H4 acetylation was determined by flow cytometry according to the protocol described elsewhere (28).

### HDAC Activity Assay

Histone deacetylases activity was determined using a commercial HDAC cell-based activity assay kit (Cayman Chemical, Ann Arbor, MI, USA) according to the manufacture’s guidelines. The HDAC activity was measured with the Wallac 1420 (PerkinElmer Life and Analytical Sciences, Turku, Finland).

### Functional Metabolic Analyses

The metabolic characteristics of moDCs were analyzed using a Seahorse XF^e^96 Extracellular Flux Analyzer (Seahorse Bioscience) as described previously (29, 30). In brief, after 48 h of pulsing, 4 × 10^4^ DCs were plated in unbuffered, glucose-free RPMI supplemented with 5% dialyzed FCS and left to rest 1 h before the assay. Subsequently extracellular acidification rate (ECAR) and oxygen consumption rate (OCR) were analyzed in response to glucose (10 mM; port A), oligomycin (1 µM; port B), fluoro-carbonyl cyanide phenylhydrazone (3 µM; port C), and rotenone/antimycin A (1/1 μM; port D) (all Sigma-Aldrich). Baseline ECAR = increase in ECAR in response to injection A. Spare ECAR = increase in ECAR in response to injection B. Baseline OCR = difference in OCR between readings following port A injection and readings after port D injection. Spare OCR is difference between basal and maximum OCR, which is calculated based on the difference in OCR between readings following port C injection and readings after port A injection.

### Human T-Cell Culture and Analysis of T-Cell Polarization

For analysis of T-cell polarization, 48 h-pulsed moDCs were cultured with allogenic naive CD4^+^ T cells for 11 days in the presence of *staphylococcal enterotoxin B* (10 pg/mL). On day 6 and 8, rhuIL-2 (10 U/mL, R&D System) was added to expand the T cells. Intracellular cytokine production was analyzed after restimulation with 100 ng/mL phorbol myristate acetate and 2 µg/mL ionomycin for a total 6 h; 10 µg/mL brefeldin A was added during the last 4 h. Subsequently the cells were fixed with 3.7% paraformaldehyde (all Sigma-Aldrich). The cells were permeabilized with 0.5% saponin (Sigma-Aldrich) and stained with PE-, FITC-, and APC-labeled antibodies against IL-4 (8D4-8), IFN-γ (25723.11) (both BD Biosciences), and IL-10 (JES3-19F1) (BioLegend), respectively. Alternatively, 1 × 10^5^ T cells were restimulated using anti-CD3 and anti-CD28 (both BD Biosciences), 24 h after restimulation, supernatants were collected, and IL-10 production by T cells was measured by ELISA (Sanquin).

### T-Cell Suppression Assay

For analysis of suppression of proliferation of bystander T cells by test T cells, 5 × 10^4^ SCFA-pulsed DCs were cocultured with 5 × 10^5^ naive CD4^+^ T cells for 6 days. These T cells (test T cells) were harvested, washed, counted, stained with the cell cycle tracking dye 1 µM Cell Trace Violet dye (Thermo Fisher Scientific) and irradiated (3,000 RAD) to prevent expansion. Bystander target T cells (responder T cells), which were allogeneic memory T cells from the same donor as the test T cells, were labeled with 0.5 µM cell tracking dye 5,6-carboxy fluorescein diacetate succinimidyl ester (CFSE). Subsequently, 5 × 10^4^ test T cells, 2.5 × 10^4^ responder T cells, and 1 × 10^3^ LPS-stimulated DCs were cocultured for 6 days. Proliferation was determined by flow cytometry, by co-staining with CD4 PE-Cy7 (clone SK3) and CD25 APC (clone 2 A3) (both BD Bioscience). To some cultures, where indicated, 10 µg/mL anti-IL-10 antibody (BioLegend), 10 µM ALK5 (Sigma-Aldrich), 10 µM DEAB, 20 ng/mL recombinant human TGF-β1 (BioLegend) (kind gift from Dr. L. Boon), or control antibody IgG1 (BioLegend) was added during the DC-T cell coculture or during the test-responder T cell coculture.

### Quantitative Real-time PCR

RNA was extracted from snap-frozen 16 h-stimulated DCs. The isolation of mRNA was performed according to the manufacturer’s instruction using RNeasy plus micro kit (Qiagen). cDNA was synthesized with reverse transcriptase kit (Promega), and PCR amplification by the SYBER Green method was done using CFX (Biorad). Specific primers for detected genes are listed in supplemental experimental procedures. Relative expression was determined using the ΔΔ*C*^t^ method.

### Small Interfering RNA (siRNA) Electroporation

On day 4 of the DC culture, the cells were harvested and transfected with either no siRNA (R buffer only, provided by Invitrogen), 20 nM control siRNA or 20 nM GPR109A siRNA (both Dharmacon) using Neon Transfection System (Invitrogen) with the following setting: 1,600 V, 20 ms width, one pulse. Following electroporation, 3.5 × 10^5^ cells were seeded per well in to a 24-well plate containing RPMI media without antibiotics. After 24 h, culture medium (RPMI) supplemented with 10% HI-FCS, rIL4 (0.86 ng/mL. R&D system Minneapolis, MN, USA) and rGM-CSF (20 ng/mL, Invitrogen, Carlsbad, CA, USA) was added. The transfection efficiency was routinely greater than 80%. *GPR109A* silencing efficiency was determined by quantitative RT PCR.

### Assay for Transposase-Accessible Chromatin with High-Throughput Sequencing (ATAC-seq) Analysis

5 × 10^4^ moDCs stimulated for 6 h with indicated reagents were subjected to ATAC-seq as described elsewhere (31). In brief, the cells were spun down immediately at 500 × *g* for 10 min at 4°C. Subsequently, the pellet was resuspended in TD Buffer (Illumina Nextera kit), transposase enzyme (Illumina Nextera kit, 15028252), and nuclease-free water in a total of 50 µL reaction for 30 min at 37°C. The DNA was then purified using Ampure XP beads (Beckman Coulter) and eluted in a final volume of 10 µL. Libraries were constructed according to Illumina protocol using the DNA treated with transposase, Kapa HiFi master mix and library-specific Nextera index primers. The following PCR condition was used to generate the libraries: 72°C, 5 min; 95°C, 2 min; (98°C, 20 s; 63°C, 30 s; 72°C, 1 min) 11× cycles; hold at 4°C. Prepared libraries went through quality control analysis using an Agilent Bioanalyzer. Samples were then used for next generation sequencing using Illumina Hiseq 2500 platform. All ATAC-seq datasets were aligned to build version NCBI37/HG19 of the human genome using Bowtie2 (version 2.2.1). For alignment and peak-calling of the ATAC-seq data the Biopet Carp pipeline was used (http://biopet-docs.readthedocs.io/en/latest/pipelines/carp/).

### Statistical Analysis

Data were analyzed for statistical significance using GraphPad Prism 7.0 statistical software (GraphPad Software, La Jolla, CA, USA). Comparison between groups was performed using the Student’s *t*-test or two-way ANOVA test. All data are shown as means ± SEM. Differences were considered significant if *p* < 0.05.

## Results

### Butyrate Suppresses LPS-Induced Activation and Metabolic Reprogramming in Human DCs

**Figure 1:**
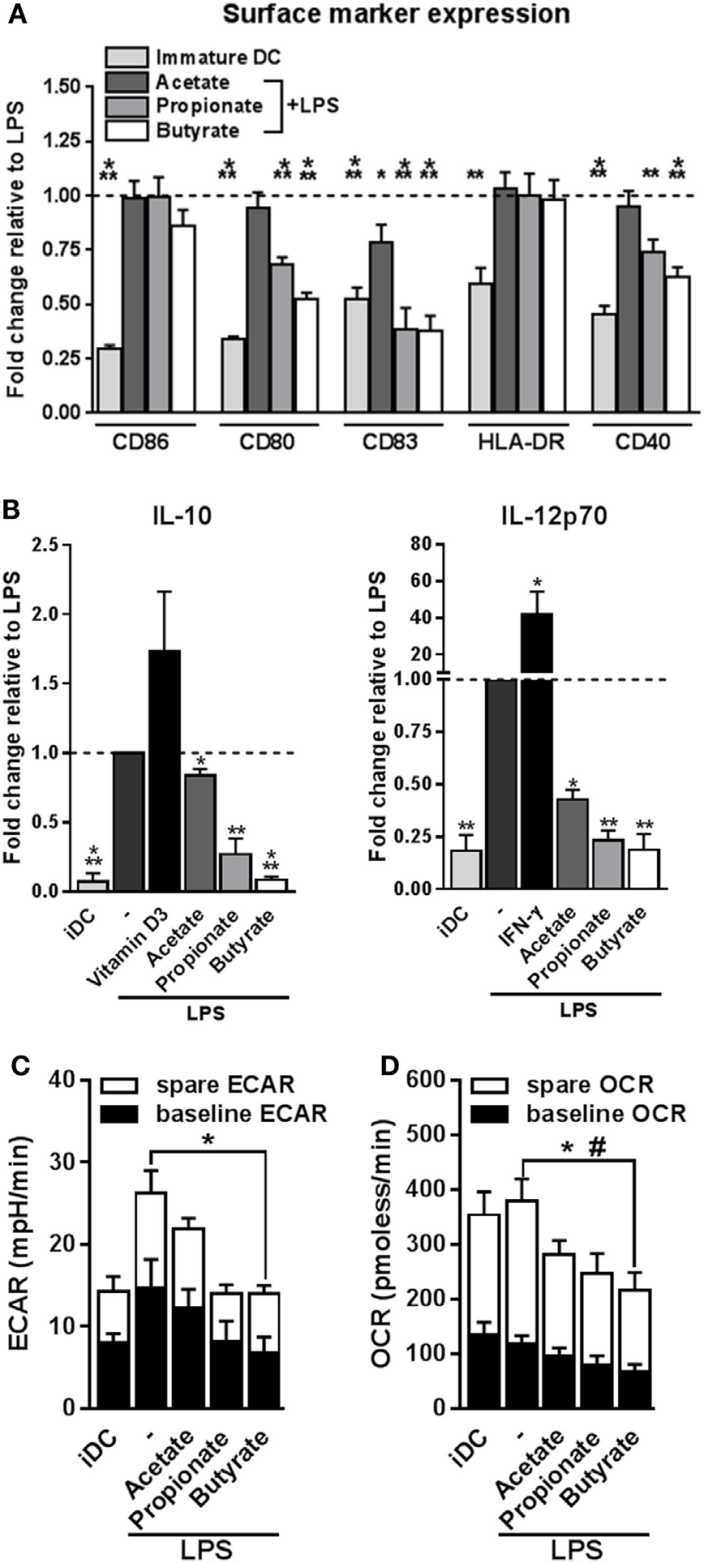
Butyrate suppresses LPS-induced activation and metabolic changes in human dendritic cells (DCs). **(A,B)** Monocyte-derived DCs were left untreated (iDC) or stimulated as indicated for 48 h after which **(A)** expression of maturation markers was analyzed by flow cytometry and **(B)** supernatants were collected and concentrations of indicated cytokines were determined by ELISA. **(A)** The expression levels of maturation markers are based on the geometric mean fluorescence and are shown relative to DCs stimulated with LPS, which was set to 1 for each marker (dashed line). **(B)** Vitamin D3 and IFN-γ-stimulated DCs were taken along as IL-10- and IL-12-inducing controls, respectively. **(C,D)** Metabolic phenotype of differently stimulated DCs was assed using a Seahorse extracellular flux analyzer. **(C)** Baseline and spare extracellular acidification rate (ECAR) were determined as described in Section “Materials and Methods,” with significant differences in baseline ECAR indicated with *. **(D)** Baseline and spare mitochondrial oxygen consumption rate (OCR) were determined as described in Section “Materials and Methods,” with significant differences in baseline or spare OCR indicated with * and ^#^, respectively. **(A–D)** Bar graphs represent means ± SEM of at least five experiments. ^*,#^*p* < 0.05, **p < 0.01, ***p < 0.001 based on paired Student’s *t*-test.

Tolerogenic compounds are often able to interfere with DC activation induced by pro-inflammatory signals. Therefore, we examined how acetate, propionate and butyrate influenced several markers of activation of human moDCs during co-stimulation with LPS, a toll-like receptor-4 ligand. Initial dose response experiments revealed that stimulation of moDCs with 2 mM SCFAs had the strongest biological effects (Figure S1A in Supplementary Material) without becoming toxic (Figure S1B in Supplementary Material). This, together with the fact that several other studies, in which the effects of SCFAs on immune cells were investigated, used a similar concentration (19, 24, 32, 33), we decided to use SCFAs at 2 mM in these and further experiments. Treatment with butyrate, and to a lesser extent with acetate and propionate, antagonized the LPS-induced upregulation of costimulatory markers CD83, CD80, and CD40 (Figure 1A). In line with this, all SCFAs lowered production of both IL-10 and IL-12 induced by LPS, with the strongest suppression induced by butyrate (Figure 1B). Given the importance of metabolic rewiring for DC activation (34) and the fact that SCFAs can act as direct substrates for several core metabolic pathways in the intestinal epithelium (23), we also analyzed the effects of SCFAs on DC metabolism. As previously reported (29), LPS stimulation enhanced the ECAR, a measure of glycolysis, of human DCs. Interestingly, while acetate and propionate showed a trend to suppress LPS-induced ECAR, we found that only butyrate significantly antagonized this response (Figure 1C). Moreover, we found that butyrate significantly reduced baseline mitochondrial OCR (Figure 1D) as well as the spare respiratory capacity of LPS-stimulated DCs (Figure 1D), together suggesting that butyrate reduces the overall metabolic activity of DCs. These findings indicate that propionate and more strongly butyrate, have the capacity to suppress LPS-induced DC activation and that butyrate additionally renders DCs metabolically less active.

### Butyrate Conditions Human DCs to Induce Tr1 Cells

**Figure 2:**
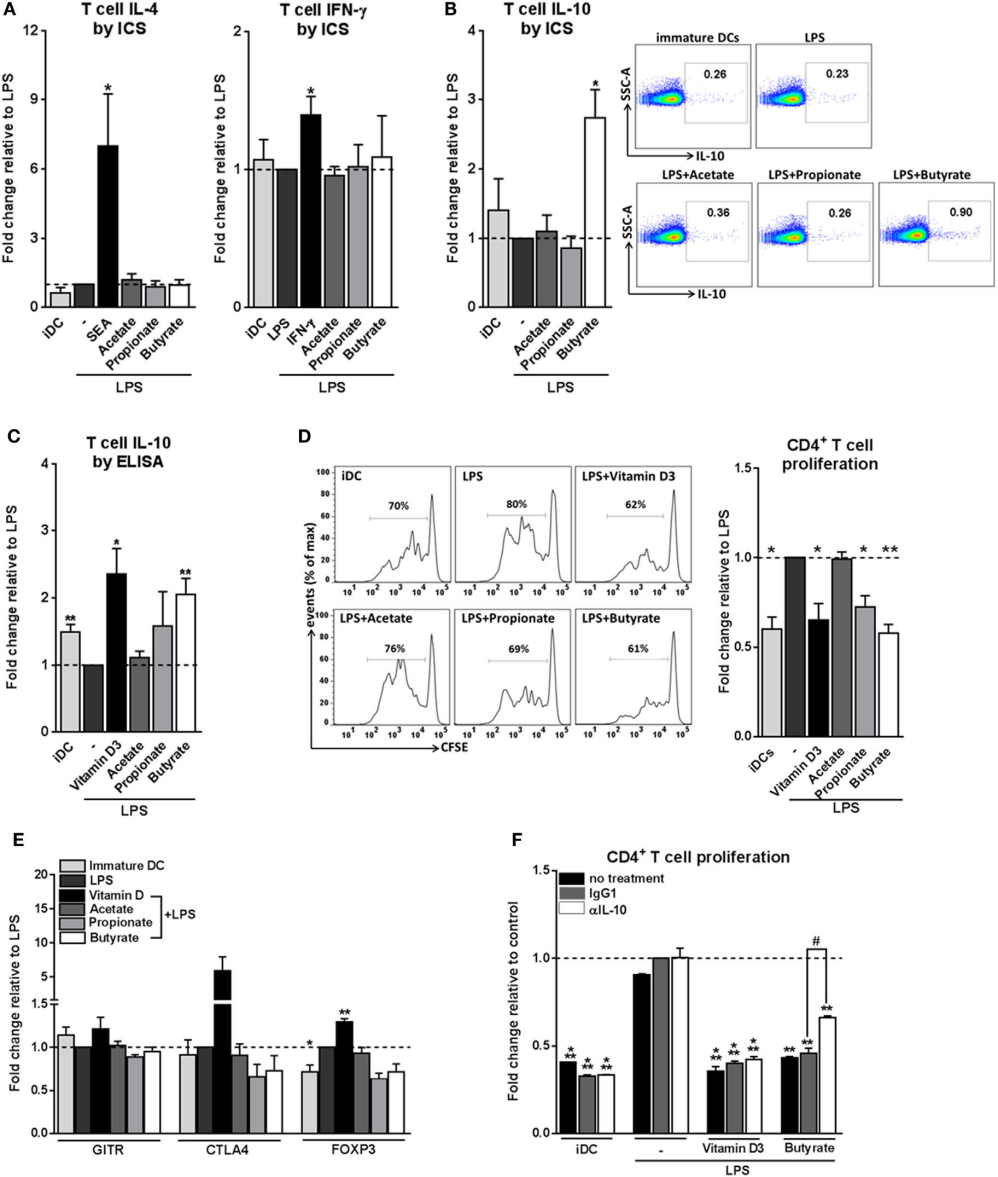
Butyrate conditions human dendritic cells (DCs) to induce type 1 regulatory T cells. **(A–C)** Differently stimulated DCs were cocultured with allogenic naive CD4^+^ T cells. After 11 days, cytokine production by T cells was analyzed **(A,B)** by intracellular cytokine staining by flow cytometry after 6 h of stimulation with PMA and ionomycin or **(C)** by ELISA after 24 h restimulation with anti-CD3/CD28. **(A–C)** Data represent fold change in panels **(A,B)** percentage of T cells that stain positive for indicated cytokines or in panel **(C)** IL-10 levels in culture supernatants, relative to data from LPS-stimulated DCs which was set to 1 for each cytokine (dashed line). **(B)** Representative flow cytometry plots are shown on the right. **(D)** T-cell suppression assay in which irradiated test T cells were cocultured with activated CFSE-labeled responder CD4^+^ T cells. On day 6, CFSE dilution of the responder T cells was assessed by flow cytometry. The left panels are representative histograms of CFSE dilution by responder T cells. Quantification of these data is shown in the bar graph and is depicted as fold change relative to data from LPS-stimulated DCs, which was set to 1 (dashed line). **(E)** Expression of regulatory markers by T cells was analyzed by flow cytometry. Bar graphs represent relative differences based on geometric mean fluorescence for glucocorticoid induced TNF receptor (GITR) and cytotoxic T-lymphocyte-associated protein 4 (CTLA4) or frequency of T cells that express forkhead box P3 (FOXP3). **(F)** For the duration of the assay as described in panel **(D)** indicated antibodies were added. Data are from one experiment representative of two, shown as means ± SEM of triplicates. **(A–E)** Bar graphs represent means ± SEM of at least three experiments. ^*,#^*p* < 0.05, **p < 0.01, ***p < 0.001 for significant differences with the control (*) or between test conditions (^#^) based on paired Student’s *t*-test.

We next set out to address how these phenotypic and metabolic changes induced by SCFAs on DCs would translate into their ability to prime T helper (Th) cell responses. To assess this, we cocultured the SCFAs-pulsed DCs with naive CD4^+^ T cells and measured the intracellular cytokine production by the T cells. We found that all three SCFAs did not condition DCs to drive Th1 or Th2 responses, since neither IFN-γ nor IL-4 production was altered (Figure 2A). Instead, we found that butyrate-stimulated DCs significantly promoted IL-10 production by T cells after restimulation with either PMA and ionomycin (Figure 2B) or anti-CD3/CD28 (Figure 2C) in a dose-dependent manner (Figure S2 in Supplementary Material). Because IL-10 is a well-known immunosuppressive cytokine released by Tregs, we next examined whether these IL-10-producing T cells were bona-fide Tregs, by determining their capacity to suppress the proliferation of other T cells. T cells that had been primed by butyrate-conditioned DCs strongly suppressed proliferation of target T cells. Propionate had a similar effect, although to a lesser extent (Figure 2D). To further characterize the phenotype of Tregs primed by butyrate-treated DCs, protein expression of three common Treg markers were measured, namely, glucocorticoid-induced TNF receptor (GITR), cytotoxic T-lymphocyte-associated protein 4 (CTLA4), and forkhead box P3 (FOXP3) (1, 35). In contrast to Tregs that were primed by DCs rendered tolerogenic by vitamin D3, Tregs induced by butyrate-stimulated DCs did not display increased expression of these markers (Figure 2E). This phenotype of a Treg with high IL-10 production but low FOXP3 expression is typical for Tr1 cells, which are defined by their dependency on IL-10 production to suppress bystander T cell proliferation (35). Indeed, when IL-10 (Figure 2F), but not transforming growth factor beta-1 (TGF-β1) (Figure S3 in Supplementary Material), was neutralized in the T cell suppressor assay, the suppressive capacity of these Tregs was significantly reduced. Together, these data demonstrate that butyrate conditions human DCs to prime IL-10-secreting Tr1 cells.

### Tr1 Cell Induction by Butyrate-Conditioned DCs Depends on RALDH1 Expression

**Figure 3:**
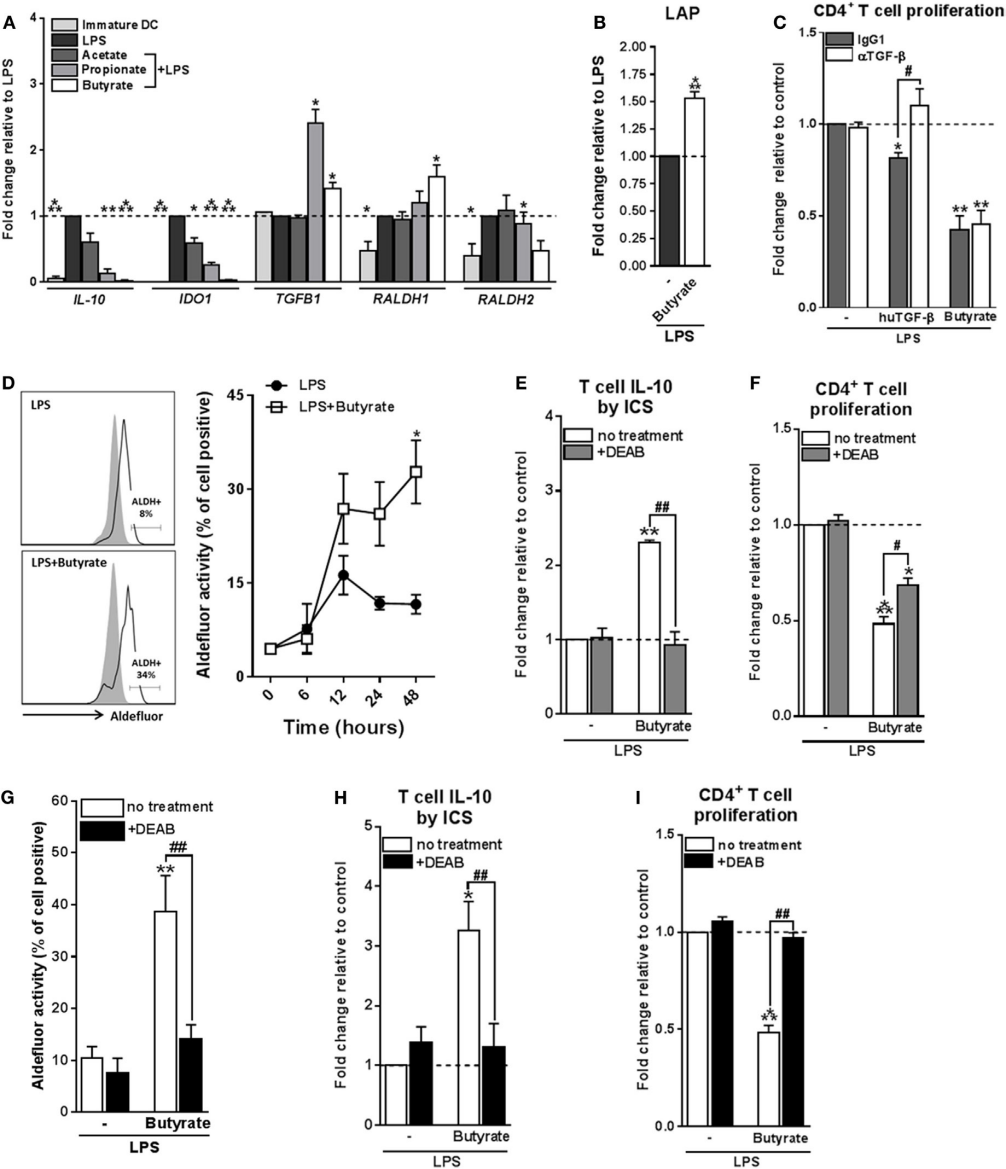
Type 1 regulatory T cell induction by butyrate-conditioned human dendritic cells (DCs) depends on retinaldehyde dehydrogenase 1 (RALDH1) expression. **(A)** mRNA expression of indicated genes was quantified using real-time qPCR of monocyte-derived DCs stimulated for 16 h. **(B)** Relative membrane bound latency-associated peptide (LAP) expression on DCs stimulated with indicated reagents as determined by flow cytometry. **(C)** T-cell suppression assay as described in Figures 2D, **(F)** blocking antibody of human TGF-β was added during the co-culture of DCs with T cells. Human TGF-β was taken along as positive control. **(D)** Retinaldehyde dehydrogenase (RALDH) activity in DCs was assessed using an Aldefluor assay with a readout by flow cytometry. Representative histograms of RALDH activity 48 h after stimulation are shown on the left, with gray shaded histograms and black lines representing DCs in which RALDH activity was assessed in the presence or absence of reversible RALDH inhibitor diethylaminobenzaldehyde (DEAB), respectively. Right graph: RALDH activity was measured at different times after stimulation and frequencies of DCs positive for RALDH activity are depicted. *p < 0.005 based on two-way ANOVA test. **(E)** IL-10 production by T cells as described in Figure 2B or **(F)** T-cell suppression assay as described in Figure 2D, but with the addition that DEAB or vehicle control was added during the DC–T cell coculture. **(G)** RALDH activity assay as described in Figure 3D, but during stimulation DEAB or vehicle control were added. **(H,I)** Same as **(E,F)**, but now DEAB was added during stimulation of DCs with LPS ± butyrate. **(A–I)** Bar graphs represent means ± SEM of at least three experiments and **(A–C,F,G,I)** are shown as fold change relative to control conditions. ^*,#^*p* < 0.05, ^**,##^*p* < 0.01, ***p < 0.001 for significant differences with the control (*) or between test conditions (^#^) based on paired Student’s *t*-test.

We next aimed to determine through which mechanism(s) butyrate-conditioned DCs prime Tr1 cells. To address this, we analyzed gene expression of several immune-regulatory factors that are known to be expressed by tolerogenic DCs and have been shown to be induced by SCFAs in immune cells, namely, *IL-10, IDO1, TGFB1*, and *RALDH1* and *RALDH2* (also known as aldehyde dehydrogenase 1 family member A1 and A2, respectively) (36) (Figure 3A). We found that *TGFB1* mRNA was upregulated by both butyrate and propionate, while *RALDH1* was selectively induced by butyrate. This prompted us to further study the role of TGF-β and RALDH1 in Tr1 cell induction by butyrate-conditioned DCs. To this end, we quantified LAP expression, which is a protein derived from the N-terminal region of the *TGFB1* gene product and binds TGF-β on the cell surface to keep it in its inactive form. In line, with the mRNA expression data, we found that the level of LAP protein expression was significantly increased by both propionate- and butyrate-stimulated DCs (Figure 3B; Figure S4A in Supplementary Material). However, while blocking of TGF-β signaling using the SMAD2/3 inhibitor ALK5 did reverse Treg induction by exogenously added TGF-β, it did not affect the Tr1-priming capacity of butyrate-conditioned DCs (Figure 3C). This suggests that butyrate does not license DCs to prime Tr1 cells through induction of TGF-β.

We next assessed the role of RALDH1. RALDH1 converts vitamin A into retinoic acid, which through the activation of retinoic acid receptor has been shown to induce tolerogenic properties in DCs as well as to directly drive Treg differentiation of T cells (37). We found that in line with the increased mRNA expression of *RALDH1*, DCs stimulated with butyrate, but not with acetate or propionate, increased the enzymatic activity of RALDH in a time-dependent manner both in the presence (Figure 3D) and absence of LPS (Figures S4B,C in Supplementary Material). To test the role of RALDH activity in Tr1 cell induction by butyrate-treated DCs, we used DEAB, a reversible inhibitor of RALDH (38) during the DC–T cell coculture. This treatment abolished IL-10 production and reduced the suppressive capacity of the T cells (Figures 3E,F), indicating that RALDH activity is a key factor expressed by DCs to promote Tr1 cells and that DC-derived RA acts on T cells to prime their regulatory properties. In addition, we wondered whether RA produced by DCs may also act in an autocrine fashion to enforce their tolerogenic potential. Blocking RA generation by DCs from the beginning of the stimulation with butyrate blunted the ability of butyrate to increase RALDH activity in these cells (Figure 3G) and, as a consequence, in the inability of these cells to promote Tr1 cells (Figures 3H,I). Together, these findings suggest that initial butyrate-driven RALDH activity by means of production of RA is required to maintain its own expression, which licenses these DCs to subsequently prime Tr1 cells.

### HDAC Inhibition by Butyrate Is Not Sufficient for Inducing tolDCs

**Figure 4:**
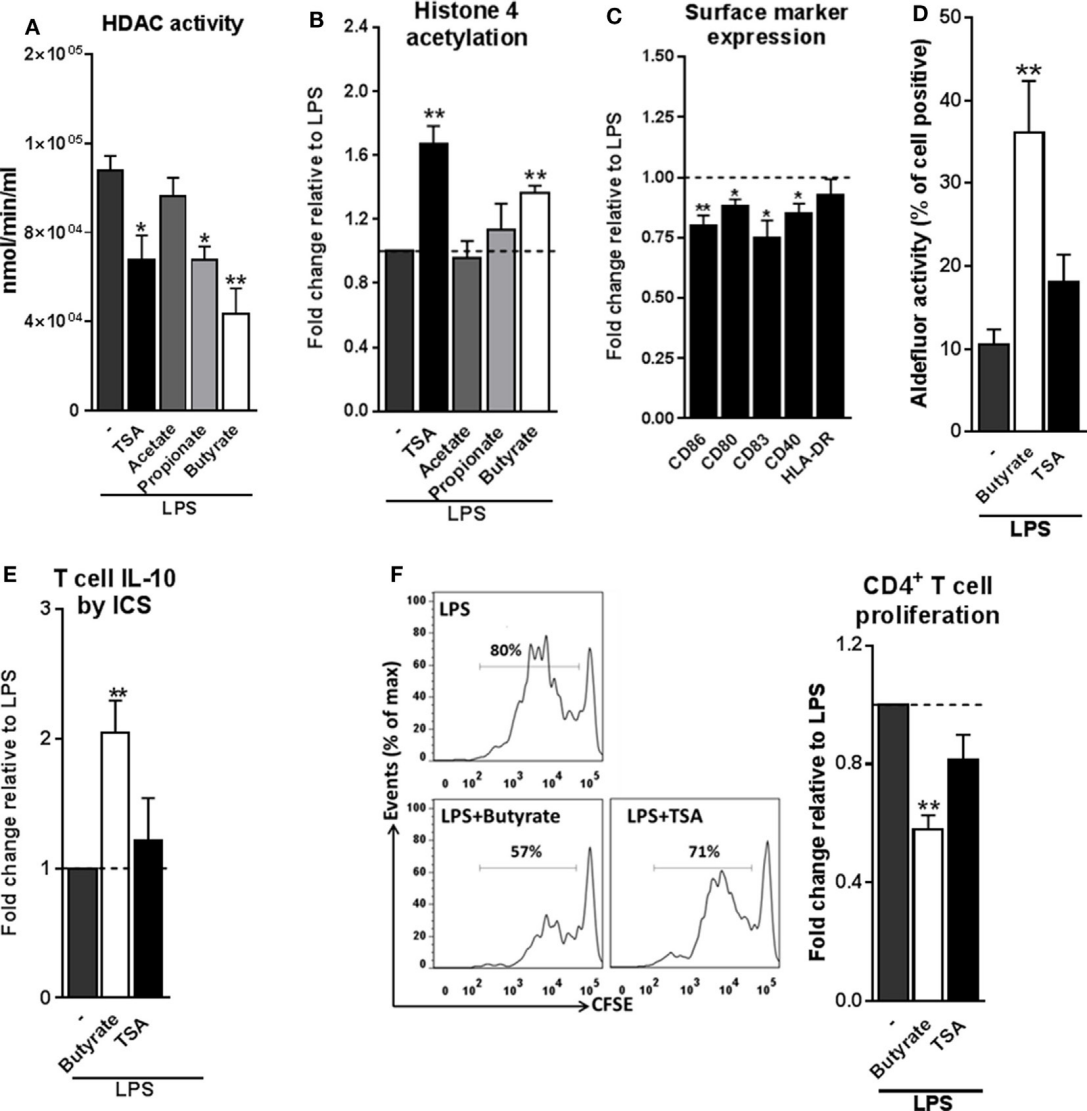
Histone deacetylases (HDAC) inhibition by butyrate is not sufficient for inducing type 1 regulatory T cells-promoting human tolDCs. **(A)** Dendritic cells (DCs) were stimulated for 24 h with indicated reagents and then assayed for HDAC activity. **(B)** Analysis of histone 4 acetylation by flow cytometry of DCs stimulated with indicated reagents for 6 h. **(C)** The expression of maturation markers of DCs stimulated for 48 h with TSA was analyzed using flow cytometry. The expression of surface marker levels is based on the geometric mean fluorescence. **(D)** Retinaldehyde dehydrogenase activity assay as described in Figure 3D. **(E)** IL-10 production by T cells as described in Figure 2B. **(F)** T-cell suppression assay as described in Figure 2D. **(A–F)** Bar graphs represent means ± SEM of at least three experiments and **(B,C,E,F)** are shown as fold change relative to control conditions. *p < 0.05, **p < 0.01, ***p < 0.001 based on paired Student’s *t*-test.

We next set out to investigate the mechanisms through which butyrate drives RALDH1 expression in human DCs. Two mechanisms have been described in other immune cells, namely, HDAC inhibition and GPR signaling (5, 11, 12, 39, 40). To first establish whether butyrate could affect HDAC activity in human DCs, we performed an HDAC activity assay on differently stimulated moDCs. As expected, TSA, a well-known HDAC inhibitor with broad specificity (20, 39), was effective in inhibiting HDAC activity in DCs (Figure 4A). Butyrate, and to a lesser extent propionate, also displayed the capacity to inhibit HDAC activity in human DCs. Importantly, this finding was corroborated by the observation that histone 4 acetylation was increased in DCs exposed to butyrate (Figure 4B). We did not see major changes in histone 3 acetylation as determined by flow cytometry (Figure S5 in Supplementary Material). We hypothesized that if HDAC inhibition would be underlying the ability of butyrate to induce tolDCs, then TSA would be able to recapitulate the effects of butyrate. Indeed, TSA suppressed LPS-induced expression of several DC activation markers (Figure 4C). However, TSA treatment only marginally promoted RALDH activity in DCs (Figure 4D) and concordantly, failed to significantly induce IL-10-producing (Figure 4E) functional Tr1 cells (Figure 4F). These data indicate that while HDAC inhibition alone is sufficient to recapitulate some of the modulatory effects of butyrate (e.g., suppression of LPS-induced maturation marker expression), it is insufficient in inducing Tr1 cells by DCs.

### Signaling through GPR109A by Butyrate Is Required but Not Sufficient for Inducing Tr1 Cell-Promoting tolDCs

**Figure 5:**
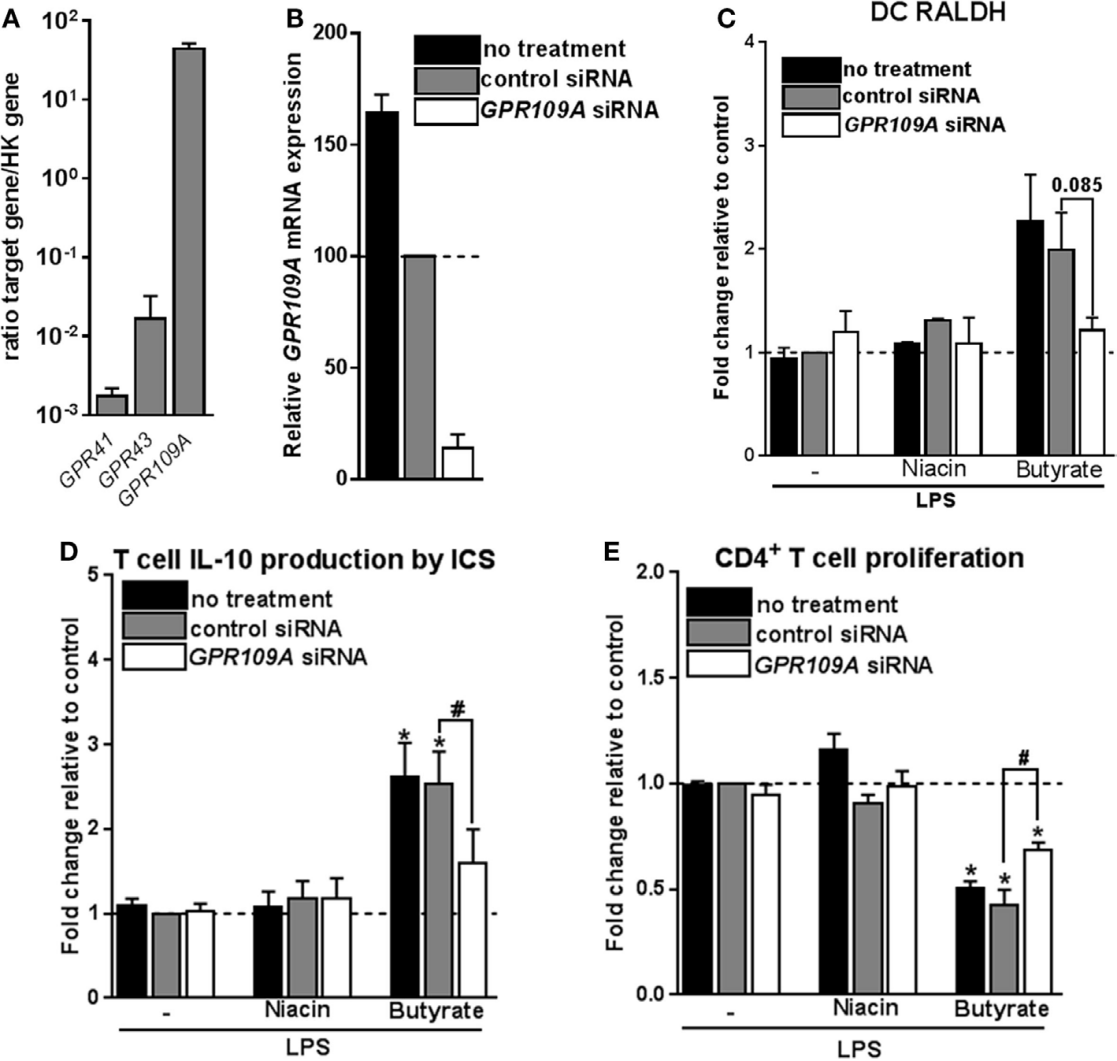
Signaling through G protein-coupled receptor (GPR) 109A by butyrate is required yet not sufficient for inducing type 1 regulatory T cells-promoting human tolDCs. **(A)** mRNA expression of indicated genes was quantified using real-time qPCR of unstimulated dendritic cells (DCs). Expression is shown relative to housekeeping gene beta-actin. **(B–E)** *GPR109A* expression was silenced by small interfering RNA (siRNA) on day 4 of DC differentiation after which **(B)** silencing efficacy was determined by real-time qPCR on day 6. **(C)** Retinaldehyde dehydrogenase (RALDH) activity induced by butyrate and GPR109A ligand niacin was assessed as described in Figure 3D. **(D)** IL-10 production by T cells as described in Figure 2B was determined, and **(E)** T-cell suppression assay was performed as described in Figure 2D. **(A)** Bar graphs represent means ± SEM of at least three experiments and **(B–E)** are shown as fold change relative to control conditions. ^*,#^*p* < 0.05, **p < 0.01, ***p < 0.001 for significant differences with the control (*) or between test conditions (^#^) based on paired Student’s *t*-test.

The inability of HDAC inhibition to induce Tr1 cell-priming tolDCs, led us to assess the role of GPRs in this process. The major GPRs activated by SCFAs are GPR41, GPR43, and GPR109A (4, 10, 11). Acetate and propionate are the most potent activators of GPR41 and GPR43, while butyrate more effectively binds to GPR109A (4, 9). Consistent with a recent report (14), we found that *GPR109A* but not *GPR41* or *GPR43* are expressed by moDCs (Figure 5A). To investigate the role of GPR109A in mediating the modulatory effects of butyrate on human DCs, *GPR109A* was silenced using siRNA, resulting in >85% silencing at the mRNA level (Figure 5B) and a corresponding loss of the ability of niacin, a natural ligand of GPR109A, to suppress LPS-induced TNF-α production (13) (Figure S6A in Supplementary Material). Silencing of *GPR109A* did not interfere with the capacity of butyrate to modulate LPS-induced DC maturation (Figure S6B in Supplementary Material). Importantly, however, we found that butyrate failed to induce RALDH activity in DCs in which *GPR109A* was silenced (Figure 5C). As a result, these DCs largely lost the ability to promote IL-10 production by T cells (Figure 5D) and functional Tr1 cells (Figure 5E). Interestingly, however, stimulation with niacin was not sufficient to promote RALDH activity in DCs nor did it enhance their ability to induce Tr1 cells. This suggests that GPR109A signaling is required yet not sufficient for human tolDC induction by butyrate.

### Butyrate Depends on the Combination of HDAC Inhibition and GPR109A Signaling to Prime Tr1 Cell-Inducing tolDCs

**Figure 6:**
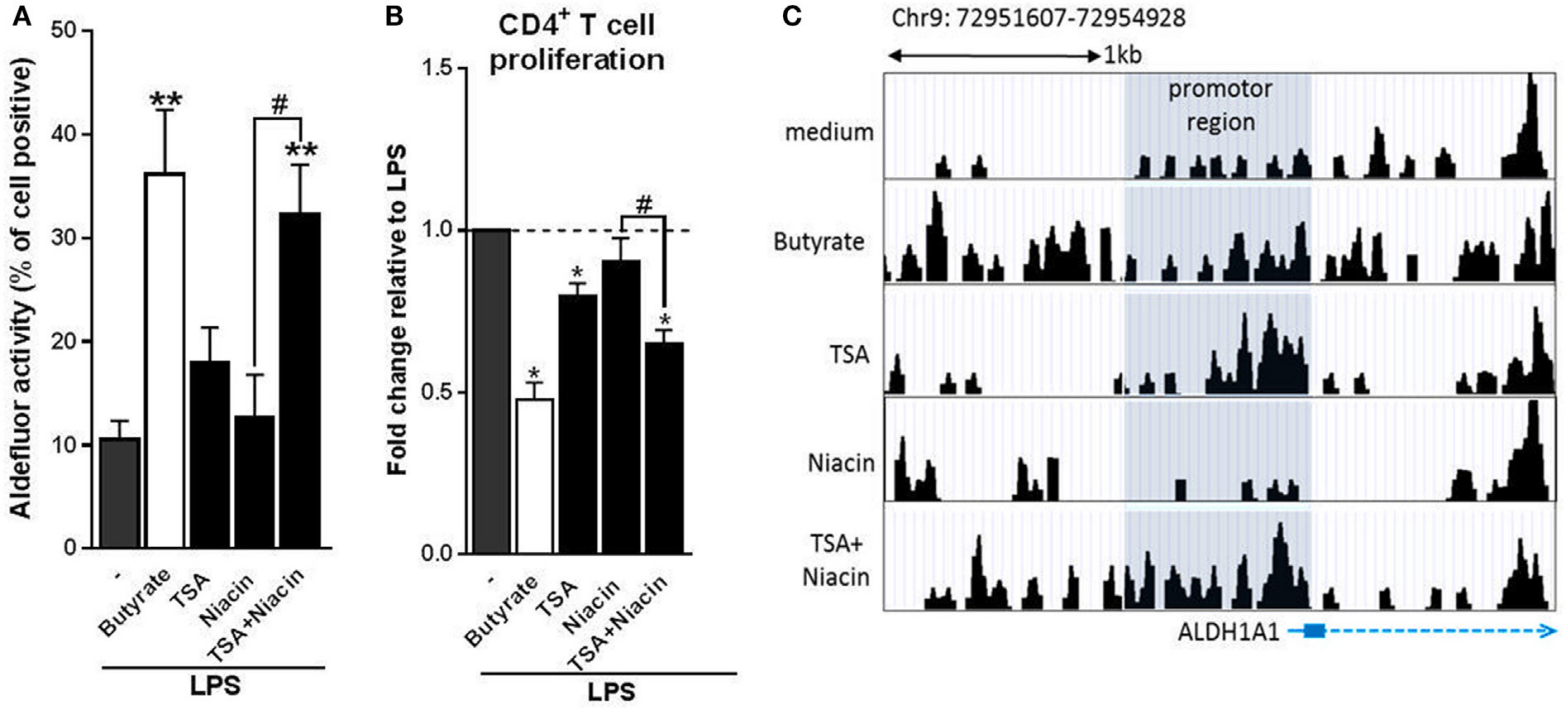
Butyrate depends on the combination of histone deacetylases inhibition and G protein-coupled receptor 109A signaling to prime type 1 regulatory T cell-inducing human tolDC. **(A)** Retinaldehyde dehydrogenase (RALDH) activity assay as described in Figure 3D. **(B)** T-cell suppression assay as described in Figure 2D. **(C)** Assay for Transposase-Accessible Chromatin with high-throughput sequencing analysis of the promoter region (highlighted in gray) of RALDH1 gene locus 6 h after stimulation of dendritic cells (DCs) with indicated reagents. **(A)** Bar graphs represent means ± SEM of at least three experiments and **(B)** are shown as fold change relative to control conditions. **(C)** Data from one of three experiments are shown. ^*,#^*p* < 0.05, **p < 0.01 for significant differences with the control (*) or between test condition (^#^) based on paired Student’s *t*-test.

Given that butyrate inhibited HDAC activity in human DCs and that it dependents on GPR109A signaling to promote tolDCs, but that neither stimulation of GPR109A signaling nor HDAC inhibition alone was sufficient for induction of tolDCs, we evaluated whether butyrate requires both HDAC inhibition and GPR109A activation for its optimal modulatory effect. To test this, we co-incubated human DCs with both HDAC inhibitor TSA and GPR109A ligand niacin. Strikingly, in contrast to the single treatments, the combinatorial treatment synergistically induced RALDH activity to a level similar to what was induced by butyrate (Figure 6A). Consistent with these findings, T cells that were primed by DCs that had been treated with the combination of TSA and niacin, displayed a stronger suppressive capacity compared to the single treatment conditions (Figure 6B). Finally, to get a better understanding of how HDAC inhibition in conjunction with GPR109A signaling would result in RALDH expression we performed an ATAC-seq analysis on the promoter region of RALDH1 to assess the level of chromatin accessibility following stimulation with butyrate, TSA, niacin or TSA in combination with Niacin. We found that TSA treatment, relative to unstimulated cells, resulted in a stronger ATAC-seq signal in the promoter region of *RALDH1*, which was comparable to the profile induced by butyrate (Figure 6C). By contrast, niacin treatment alone did not lead to opening of the chromatin in this locus, nor did it significantly alter the ATAC-seq profile induced by TSA. These findings point to two distinct roles of HDAC inhibition and GPR109A signaling in driving RALDH1 expression. Together with the observation that only the combined treatment with TSA and niacin significantly induced RALDH activity in DCs, this suggests that HDAC activity is needed for opening of the chromatin of the locus encoding RALDH1, while GPR109 signaling is required for initiation of transcription once the locus is accessible for transcription factors. Taken together, our data suggest that butyrate depends on both HDAC inhibition as well as GPR109A signaling to efficiently drive RALDH1 expression and to promote an anti-inflammatory phenotype in human DCs.

## Discussion

**Figure 7:**
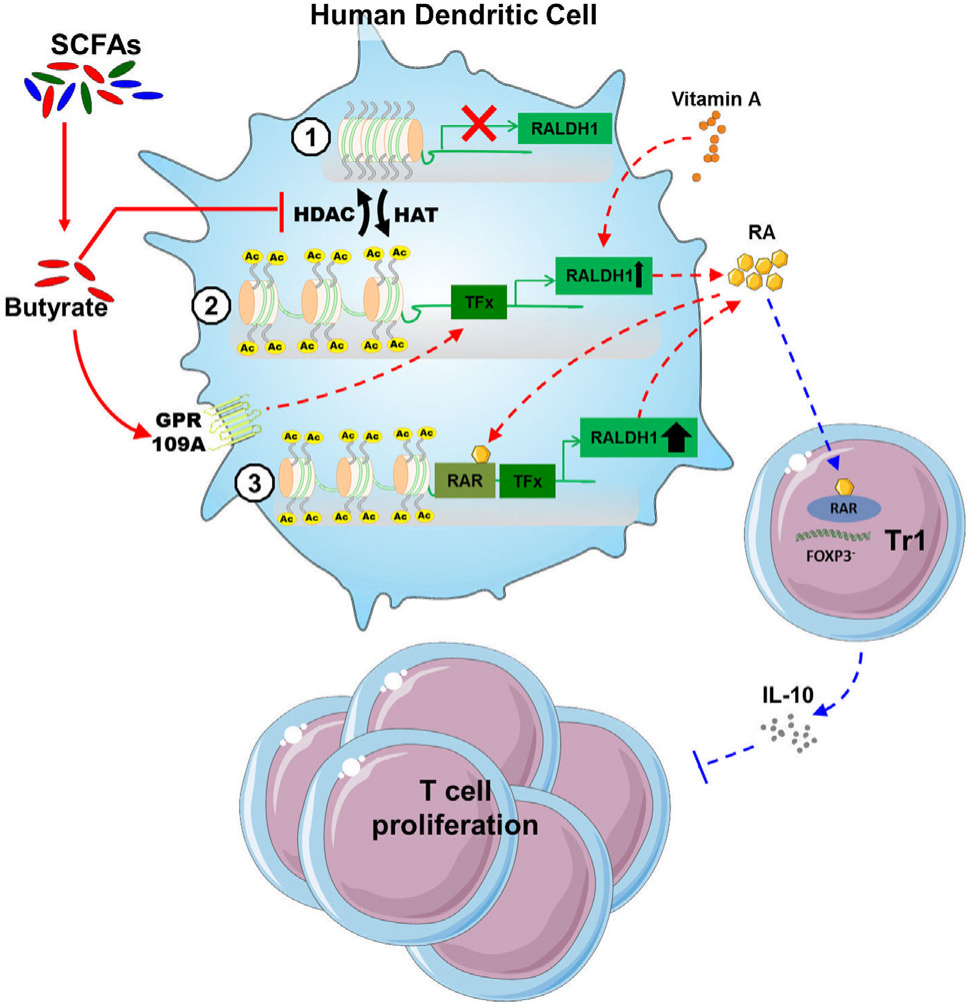
Proposed model of how butyrate conditions human dendritic cells (DCs) to prime type 1 regulatory T cells (Tr1). (1) Butyrate inhibits histone deacetylases (HDAC) activity in DCs to enhance net histone acetylation, resulting in opening of the gene locus of retinaldehyde dehydrogenase 1 (RALDH1) in human DCs. (2) The now open promoter region of RALDH1 enables butyrate through signaling *via* G protein-coupled receptor (GPR) 109A to promote transcription and expression of RALDH1. (3) This initial RALDH1 expression results in retinoic acid (RA) synthesis that further reinforces RALDH1 expression. Butyrate-induced RALDH1 expression endows human DCs with the capacity to prime IL-10-producing type 1 regulatory T cells.

Short-chain fatty acids produced by commensal bacteria, such as butyrate, have been well documented to promote anti-inflammatory responses through the modulation of various immune cells such as neutrophils, DCs, macrophages and T cells. This has been described to occur particularly in tissues that are in close contact with the lumen of the gut, where SCFAs concentrations are known to exceed 20 mM (41). Since gut-associated DCs have been shown to probe for antigens in the lumen by extending dendrites into the intestinal lumen (42), particularly DCs are likely to be exposed to high concentrations of immunomodulatory SCFAs. However, the effects of SCFAs on human DC phenotype and function have not been studied in detail. Here we find that SCFAs, in particular butyrate, suppresses LPS-induced activation and licenses them to prime functional Tr1 cells. Mechanistically, we provide evidence that butyrate, through the concerted action of both GPR109A activation and HDAC inhibition, drives the induction of RALDH1 expression and activity in human DCs. The resultant RA production on the one hand acts in autocrine manner to reinforce RALDH expression and maintain the tolerogenic properties of the DCs themselves, and on the other hand acts in a paracrine manner on T cells to differentiate them into regulatory IL-10-producing Tr1 cells (Figure 7).

Our observation that SCFAs, especially butyrate, downregulate LPS-induced expression of DC activation markers as well as cytokines released by DCs is consistent with earlier studies in murine DCs and more recently human DCs (14, 43, 44). This effect is likely to be in part mediated by the ability of butyrate to interfere with LPS-induced translocation of NF-κB, which has been described in myeloid cells before (44, 45). Here, we additionally find that this inhibitory effect of butyrate on LPS-driven changes in DC biology can be extended to cellular metabolism, by showing that butyrate lowers activity of core metabolic pathways, i.e., glycolysis and oxidative phosphorylation (OXPHOS), in human DCs. Since LPS-induced glycolysis, which occurs independently of NF-κB signaling is known to be crucial for DC activation (34), it is possible that one of the mechanisms through which butyrate interferes with LPS-driven DC activation is *via* modulation of DC metabolism. It remains to be determined how butyrate affects metabolism in DCs, but it is interesting to consider that SCFAs including butyrate can directly act as substrates for core metabolic pathways as has been well documented in intestinal epithelial cells (5, 11). In addition, butyrate has been shown to affect hypoxia-induced factor, which is a transcription factor that among others is transcriptional regulator of glycolytic enzymes (23). This low metabolic activity of butyrate-conditioned tolDCs appears to be different from what has been described for DCs that were rendered tolerogenic by vitamin D3, which were found to be metabolically characterized by increased glycolysis and OXPHOS (46, 47), suggesting that not all tolerogenic DCs share a common metabolic signature.

Several studies have demonstrated that SCFAs, in particular butyrate, are potent inducers of Tregs through the functional modulation of murine DCs (13, 24). While butyrate was recently reported to suppress pro-inflammatory cytokine expression of human DCs, the consequence of this in terms of T-cell polarization or Treg induction remained unclear. We now show that butyrate-conditioned human DCs promote the *de novo* induction of Tregs from naive T cells. Specifically, we found that butyrate-exposed DCs prime IL-10 secreting Tr1 cells. However, it should be noted that IL-10 neutralization did no completely block their suppressive effect in our model, suggesting that additional mechanisms are involved. Our findings are in line with study of Jeon et al. who found that murine colonic DCs, when exposed to butyrate, also promote Tr1 cells differentiation (48). Given that butyrate can also directly act on T cells to favor differentiation of Foxp3^+^ Tregs (24), it is likely that Tregs induced by butyrate *in vivo* through both DC-dependent and independent pathways are comprised of different subsets that mediate their immune-regulatory effects through a number of different mechanisms.

Mechanistically, our data reveal that specifically induction of *RALDH1* expression and activity is the key mechanism through which butyrate-conditioned human DCs prime Tr1 cells. RALDH enzymes are necessary for RA production by DCs from retinol (vitamin A) (49). The role of DC-derived RA in promoting Tregs responses has been well documented, especially in the gut in both mouse (50) and human DC models (37). However, the link between butyrate and the induction of RALDH1 expression in DCs has only been recently made. In this respect, our observation is in line with a recent study showing that the expression of *RALDH1* gene is induced by butyrate in human moDCs (14). However, its role in T-cell priming was not assessed. In addition, consistent with our *in vitro* findings with human DCs, high dietary fiber intake and butyrate synthesis have been linked to increased activity of RALDH in murine intestinal CD103^+^ DCs, which was found to be important for protection against colitis (13) and food allergy (51). A striking observation was that blocking of the enzymatic activity of RALDH in DCs during exposure to butyrate resulted in loss of butyrate-induced RALDH activity and Tr1-inducing ability, suggesting that RALDH-derived RA acts in an autocrine loop on DCs to reinforce their own RALDH activity required to maintain their tolerogenic potential. We additionally found that inhibition of RALDH activity during DC–T cell co-culture also reduced their Treg priming ability, implying that RALDH-derived RA subsequently acts as a key signal from DCs to differentiate naive T cells into Tregs. Independent support for this model comes from a recent study showing that treatment of moDCs with RA itself is indeed sufficient to induce RALDH expression and to endow these cells with the capacity to induce IL-10-producing Tregs in an RA-dependent manner (37).

Two of the most well-studied mechanisms through which butyrate has been shown to modulate immune cell function are inhibition of HDAC activity and signaling through GPRs (4, 5, 9, 10, 18). Our data suggest that butyrate depends on both mechanisms together to efficiently induce RALDH expression and promote functional tolDCs. This is based on the following observations: (1) silencing of *GPR109A*, resulted in the inability of butyrate to drive RALDH activity in DCs and to license them to induce Tr1 cells; (2) yet signaling through this receptor induced by a GPR109A ligand, niacin could not recapitulate the tolerogenic effects of butyrate; (3) likewise, TSA, a general HDAC inhibitor, failed to do so as well; and (4) only simultaneous treatment of DCs with niacin and TSA could functionally mimic the effects of butyrate. Other studies, using murine models, have either highlighted a role for signaling *via* GPR109A or a role for inhibition of HDAC activity in the ability of butyrate to promote tolDCs (13, 24). However, to the best of our knowledge, we now for the first time show that both modes of action are equally important and act in concert to drive RALDH expression and a tolerogenic phenotype in human DCs. These findings, together with the ATAC-seq data, lead us to speculate that inhibition of HDAC activity drives the opening of the chromatin encoding RALDH1, while concurrent signaling *via* GPR109A promotes the activation of transcription factors that then can efficiently access the promoter region of this gene to drive transcription of RALDH1. The model of how we propose this epigenetic change precedes and underpins the phenotypic change in DCs is shown in Figure 7. Further studies are warranted to identify which transcription factors downstream of GPR109A would mediate *RALDH1* expression.

In summary, we found that human DCs treated with butyrate acquire a tolerogenic phenotype, which is dependent on RALDH activity driven by the combined action of HDAC inhibition and GPR109A signaling. Our findings provide key new mechanistic insights into the immunomodulatory effects of SCFAs on human cells and highlight the importance of a well-balanced composition of our gut microbiota with sufficient SCFA-generating genera to ensure maintenance of an immune tolerant state. In addition, in line with the well documented therapeutic potential of SCFAs for a wide range of diseases (2, 4, 8, 9, 11), our work could spur the design of targetable drugs that exploit the synergetic effect of GPR109 signaling and HDAC activity in DCs to favor tolerogenic responses to treat inflammatory disorders.

## Ethics Statement

Human monocytes and T cells were obtained from blood that was donated to the Bloodbank (Sanquin, Amsterdam) by healthy volunteers. The donated material was processed and analyzed anonymously. As such, not ethical approval was required for these studies.

## Author Contributions

MK, LP, AH, and BE designed and performed experiments. MK, MY, and BE wrote the manuscript. MY and BE conceptualized and supervised the study.

## Conflict of Interest Statement

The authors declare that the research was conducted in the absence of any commercial or financial relationships that could be construed as a potential conflict of interest.
