# Supplementary material for: Butyrate Conditions Human Dendritic Cells to Prime Type 1 Regulatory T Cells via both Histone Deacetylase Inhibition and G Protein-Coupled Receptor 109A Signaling
Source: Front Immunol. 2017 Oct 30;8:1429. doi: 10.3389/fimmu.2017.01429 (PMC5670331; doi:10.3389/fimmu.2017.01429)

**Supplemental Material**

**Supplementary table 1. Primer sequences used for RT-qPCR**

**Figure S1. Butyrate suppresses LPS induced maturation of human DCs in a dose dependent manner. (a)** Monocyte-derived DCs were left untreated (iDC) or stimulated with butyarate as indicated for 48 h after which expression of maturation markers was analysed by flow cytometry. (**b**) MoDCs were left untreated or stimulated with different SCFAs at 2mM for 48 h after which cell death was determined by staining for 7AAD. Bar graphs represent means ± SEM of at least 3 experiments and (**a**) are shown as fold change relative to control condition.

**Figure S2. Butyrate conditions human DCs to prime IL-10 producing T cells in a dose dependent manner.** DC-T cell co-culture assay as described in Fig. 2A. Bar graphs represent means ± SEM of 3 experiments and are shown as fold change relative to control condition.

**Figure S3. Suppression of proliferation by T cells that are differentiated by butyrate-conditioned DCs is independent from TGF-β signaling.**

T cell suppression assay as described in Fig. 2D. Blocking antibody against TGF-β or IgG1 control antibody was added during the DC-T cell co-culture. Bar graphs represent means ± SEM of two experiments and are shown as fold change relative to control condition.

**Figure S4. LAP expression and RALDH activity of differently stimulated DCs.**

(**a**) Surface LAP expression on stimulated DCs was determined as described in Fig. 3B. (**b** and **c**) RALDH activity was assessed in stimulated DCs as described in Fig. 3D and 3E. In (**b**) the kinetic of RALDH activity was assessed on iDC and butyrate-stimulated DCs (in the absent of LPS) at 0, 6, 12, 24 and 48 h after stimulation and in (**c**) RALDH activity was measured on 6 h-differently stimulated DCs. (**b**) Bars represent means ± SEM, statistically significance of different time points per condition compared to 6 h time point. *p<0.05, **p<0.01 based on two-way ANOVA test. (**a** and **c**) Bar graphs represent means ± SEM of at least three experiments and (**a**) are shown as fold change relative to control conditions. **p<0.01, ***p<0.001 based on paired student’s T-test.

**Figure S5. Histone 3 acetylation in differently stimulated DCs.**

Analysis of histone 3 acetylation by flow cytometry of DCs stimulated with indicated reagents for 6 h. Bar graphs represent means ± SEM of at least three experiments and are shown as fold change relative to LPS control which is set to 1 (dashed line).

**Figure S6. Effect of *GPR109A* silencing on niacin and butyrate conditioned-DCs.**

*GPR109A* expression was silenced by small interfering RNA (siRNA) on day 4 of DC differentiation after which (**a**) the ability of niacin, as a natural ligand of GPR109A, to suppress LPS-induced TNF-α production was determined in supernatants by ELISA. (**b**) Expression of maturation markers of stimulated DCs was analysed by flow cytometry. (**a** and **b**) Bar graphs represent means ± SEM of at least three experiments and are shown as fold change relative to control conditions. *p<0.05, ***p<0.001 based on paired student’s T-test.

**Supplementary Table 1. Primer sequences used for RT-qPCR**

| **Gene** | **Forward (5’-3’)** | **Reverse (5’-3’)** |
| --- | --- | --- |
| *ACTB* | GCTACGAGCTGCCTGACGG | CAGCGAGGCCAGGATGGAGCC |
| *IL10* | ACCTGCCTAACATGCTTCGAG | CCAGCTGATCCTTCATTTGAAAG |
| *IDO1* | GGTCTGGTGTATGAAGGGTTCTG | GAGGAACTGAGCAGCATGTCCT |
| *TGFB1* | CCCAGCATCTGCAAAGCTC | GTCAATGTACAGCTGCCGCA |
| *RALDH1* | TGGCTTATCAGCAGGAGTGT | ACCGTACTCTCCCAGTTCTCTTC |
| *RALDH2* | GAGCAGGGTCCCCAGATTGA | CCCAGTCCTTTGCCTCCACA |
| *GPR41* | TCTCAGCACCCTGAACTCCT | TTCTGCTCCTTCAGCTCCAT |
| *GPR43* | GCCTGGTGCTCTTCTTCATC | AGGTGGGACACGTTGTAAGG |
| *GPR109* | TGCCGCCCTTCCTGATGGACA | TGTTCAGGGCGTGGTGGGGA |

**Figure S1**

**
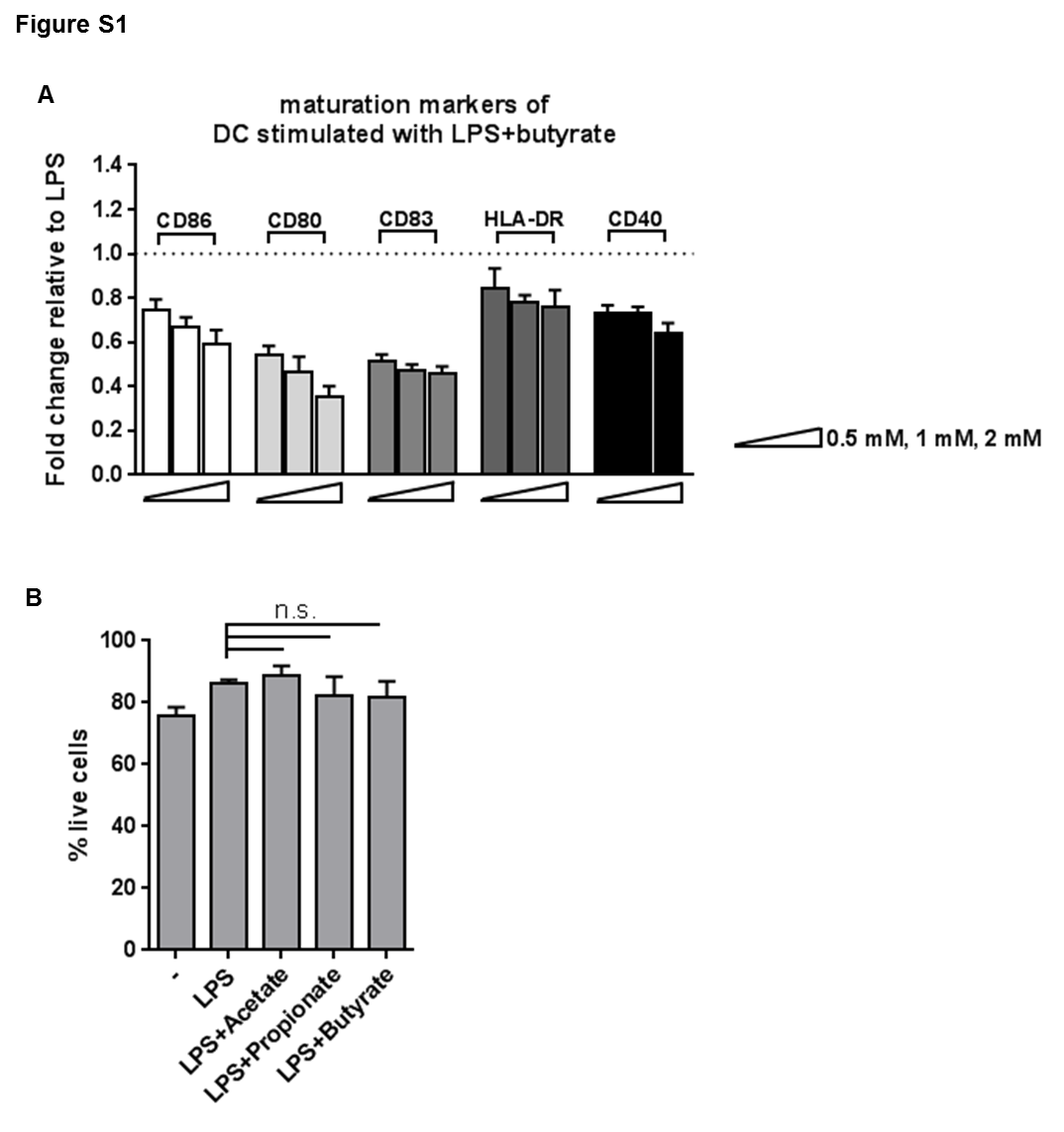
**

**Figure S2**

**
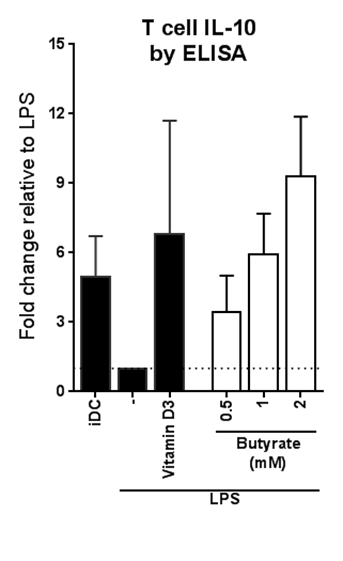
**

**Figure S3**
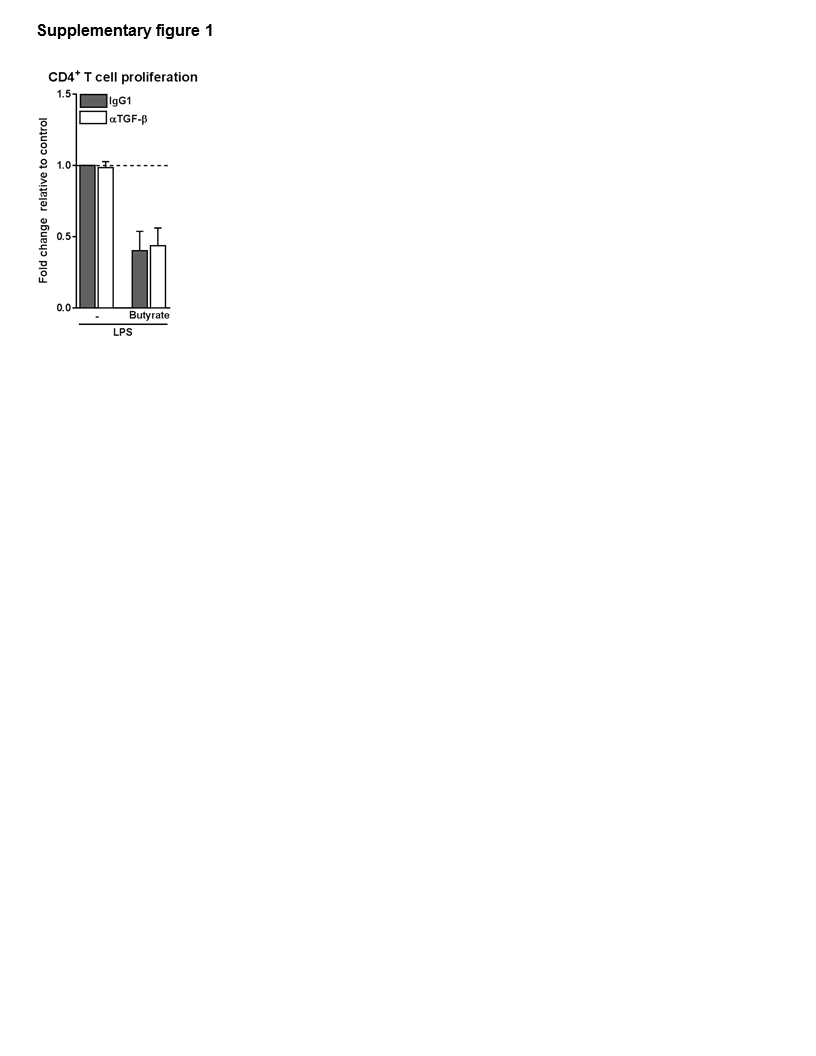


**Figure S4**
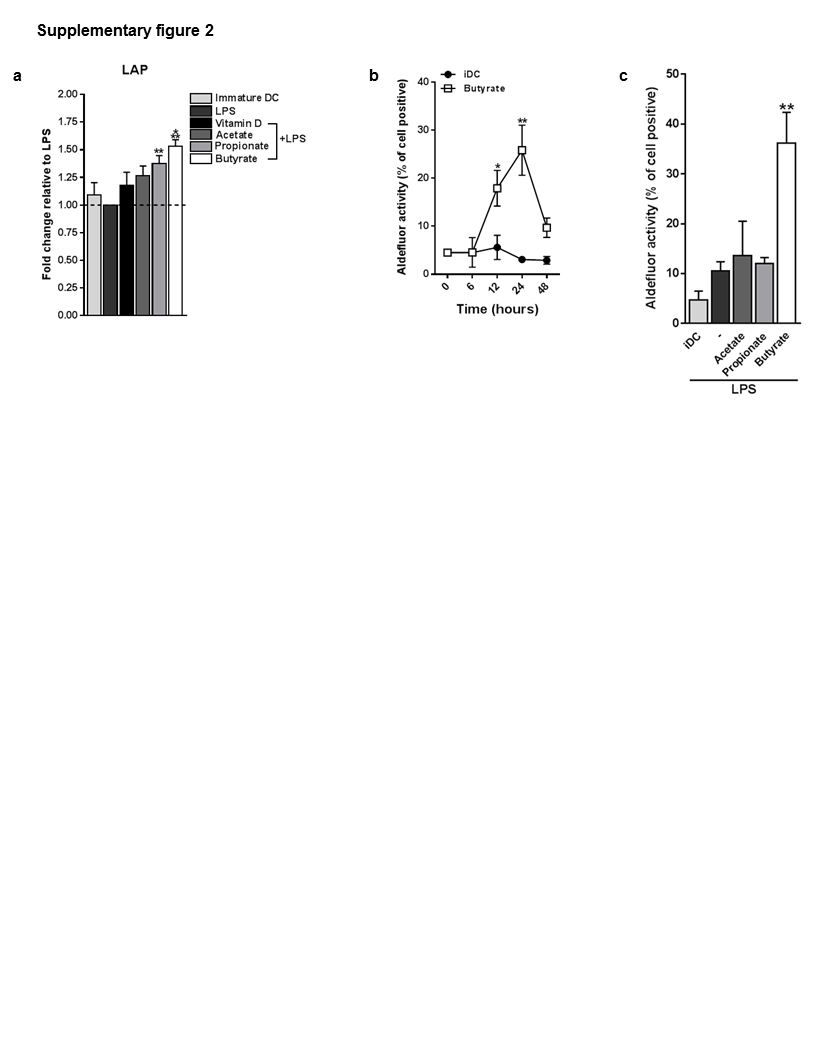


**Figure S5**
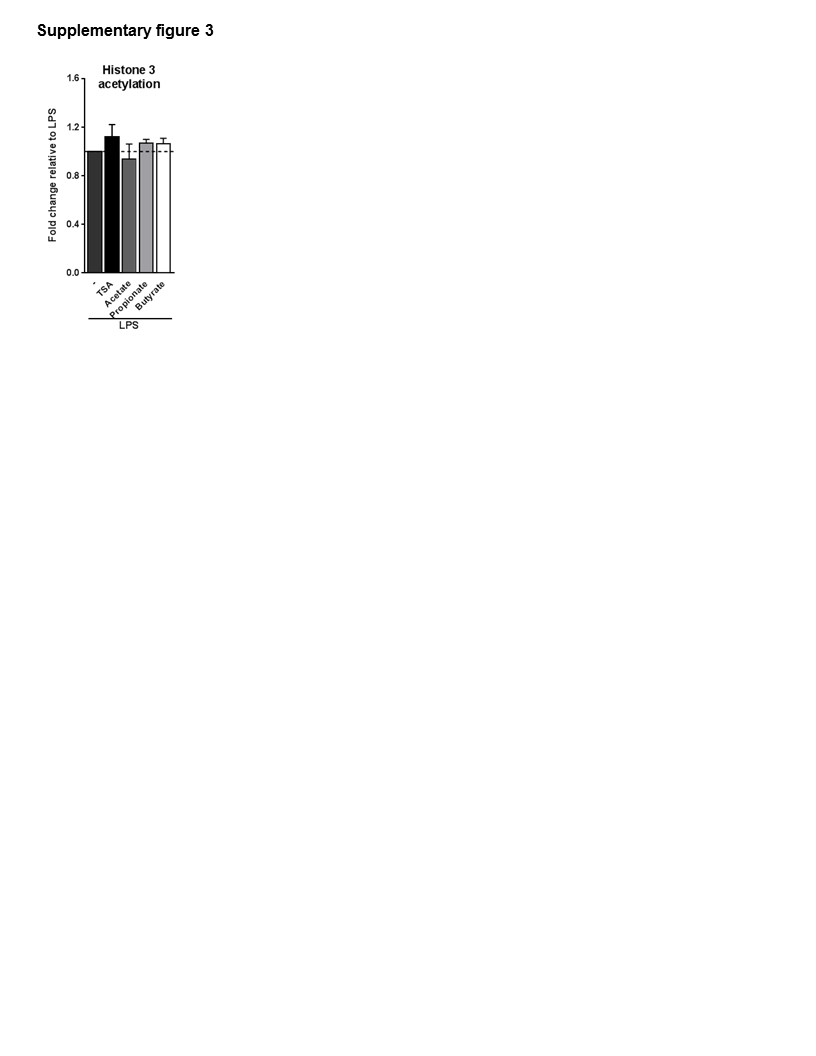


**Figure S6**
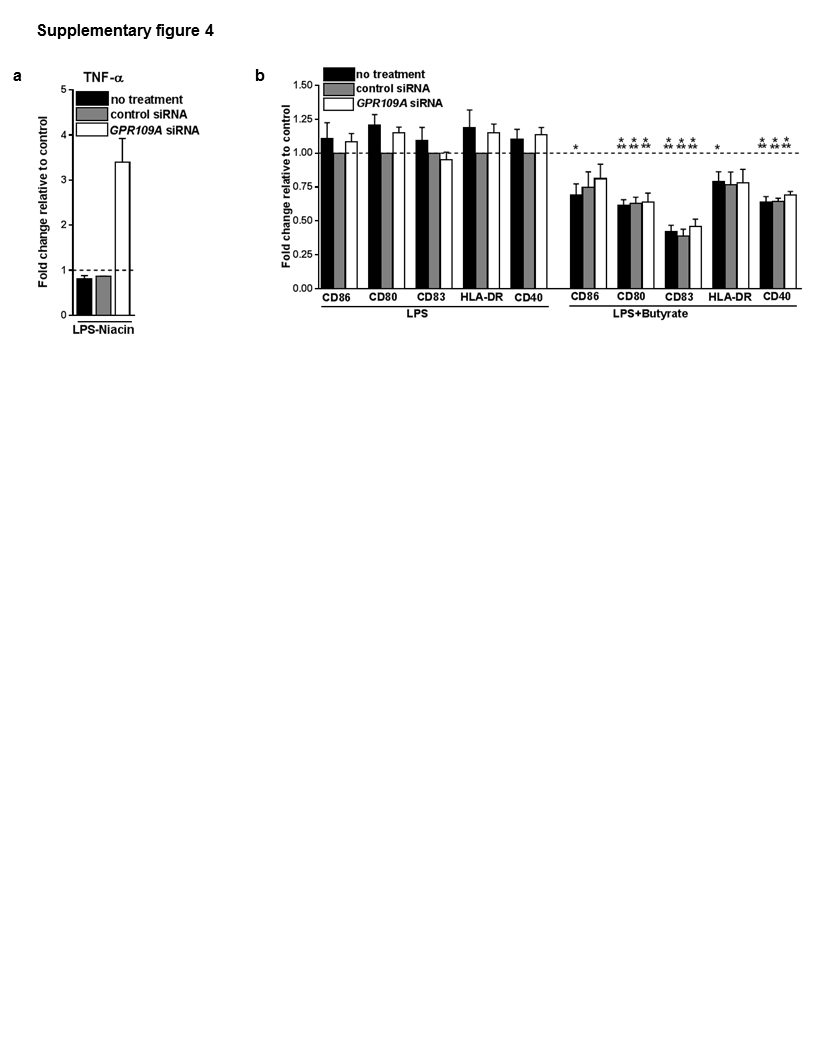

Supplement: Supplementary file 1 [file Data_Sheet_1.DOCX]
